# Intracardiac Echocardiography and Ablation of Atrial Fibrillation in Dextrocardia

**DOI:** 10.1111/anec.70030

**Published:** 2024-11-05

**Authors:** Shuang Zhang, Mingxian Chen, Lin Hu, Hanze Tang, Liyi Liao, Xuping Li

**Affiliations:** ^1^ Department of Cardiovascular Medicine The Second Xiangya Hospital of Central South University Changsha Hunan China

**Keywords:** ablation, arrhythmia, atrial fibrillation, dextrocardia

## Abstract

**Background:**

Dextrocardia with atrial fibrillation (AF) complicates radiofrequency ablation treatment.

**Methods:**

A case of successful AF ablation in dextrocardia, guided by intracardiac echocardiography (ICE) and the Carto 3 high‐density mapping system, is reported.

**Results:**

ICE‐guided transseptal puncture and three‐dimensional mapping facilitated successful pulmonary vein isolation (PVI). The patient had a good recovery with no recurrence.

**Conclusion:**

ICE and Carto 3 system's high‐density mapping aid in ablation for abnormal cardiac anatomy, reducing surgical complications.

## Graphical abstract

A patient with dextrocardia and atrial fibrillation successfully underwent radiofrequency ablation under the guidance of intracardiac echocardiography.

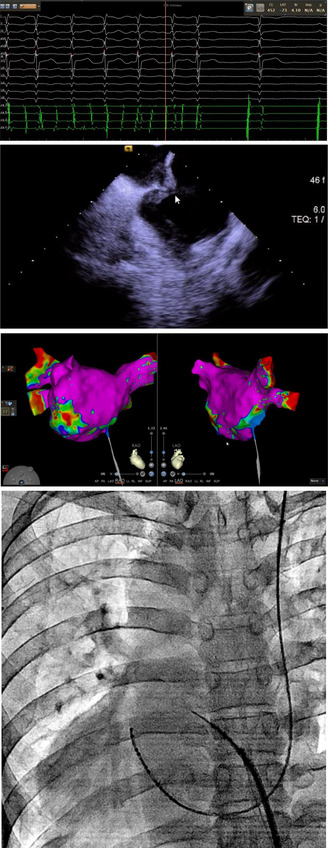

## 1 Introduction

Atrial fibrillation (AF) is one of the most common arrhythmias, and catheter ablation has gradually become a preferred treatment approach (Joglar et al. 2024). And it is gradually being applied to the treatment of atrial fibrillation in patients with congenital heart disease (Hu et al. 2023). However, treating patients with rare anatomical variations, such as dextrocardia, presents additional challenges due to the complex cardiac anatomy. Dextrocardia refers to the heart being located in the right thoracic cavity and is often accompanied by other visceral organ mirror‐image placements. This positional anomaly complicates conventional cardiac ablation techniques (Chen et al. 2023).

The unique cardiac anatomy of dextrocardia makes precise localization and ablation challenging using traditional methods. The integration of intracardiac echocardiography (ICE) and three‐dimensional mapping systems (such as Carto 3) offers new perspectives and technical solutions for ablation in these complex scenarios.

This case report illustrates the successful radiofrequency ablation of a patient with AF and dextrocardia, utilizing ICE in conjunction with the Carto 3 high‐density mapping system. By detailing the procedural steps, technical highlights, and postoperative follow‐up results, this report aims to provide clinicians with insights and references for exploring new methods and technologies for AF treatment in patients with complex cardiac anatomy.

## 2 Case Presentation

The patient is a male who presented with recurrent palpitations. He has no history of major illnesses. Holter monitoring indicated paroxysmal AF with a maximum heart rate of 176 beats per minute. The patient experienced palpitations during rapid heart rates. Despite taking oral β‐blockers, the reduction in palpitations was not significant. The diagnosis was confirmed as paroxysmal AF. Transesophageal echocardiography (TTE) revealed dextrocardia, with normal heart chamber size and function.

## 3 Electrophysiological Procedure

**FIGURE 1:**
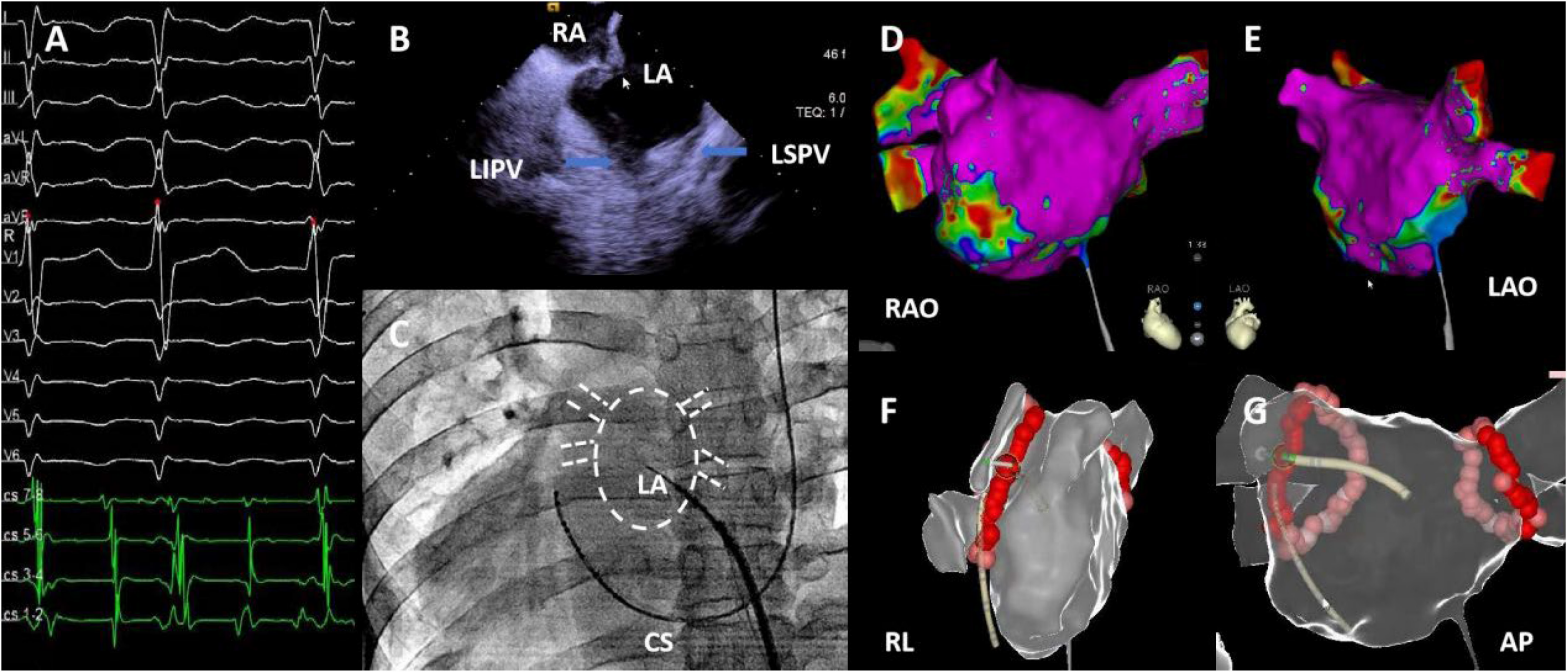
A: Electrophysiology study shows atrial stimulation inducing arrhythmia; surface and intracardiac electrograms indicate atrial fibrillation. (B) Intracardiac echocardiography image showing the puncture sheath (white arrow) aligned with the atrial septum, positioned centrally (LA, left atrium; RA, right atrium; LSPV, left superior pulmonary vein; LIPV, left inferior pulmonary vein). (C) X‐ray in the anterior–posterior view showing the transseptal puncture needle passing through the fossa ovalis into the left atrium (CS: coronary sinus electrode). (D) Right anterior oblique (RAO) 30° three‐dimensional reconstruction model with substrate mapping (purple indicates healthy atrial substrate). (E) Left anterior oblique (LAO) 45° three‐dimensional reconstruction model with substrate mapping. (F) Right lateral (RL) three‐dimensional reconstruction model showing circumferential pulmonary vein ablation points (red dots indicate ablation sites). (G) Anterior–posterior (AP) three‐dimensional reconstruction model showing circumferential pulmonary vein ablation points (red dots indicate ablation sites).

During the electrophysiological study, AF was induced using atrial S1S1 stimulation at 240 ms (Figure 1A). Due to the patient's dextrocardia, the cardiac orientation was unexpected, making transseptal puncture under X‐ray guidance challenging. The use of ICE allowed for a clear view of the puncture site on the atrial septum. Under ICE guidance, the puncture sheath was aimed at the right‐sided pulmonary veins (Figure 1B). Fluoroscopy in the anteroposterior view confirmed the entry of the puncture sheath into the left atrium (Figure 1C).

Subsequently, the Carto 3 high‐density mapping system (Biosense Webster) was used to reconstruct a three‐dimensional model of the left atrium (Figure 1D,E). The three‐dimensional mapping revealed a clear mirror‐image dextrocardia, with the right anterior oblique 45° view corresponding to the usual left anterior oblique 45° view and the left anterior oblique 30° view corresponding to the usual right anterior oblique 30° view. Pulmonary vein isolation was then performed using saline irrigation (STSF, 50 W, at 18 mL/min during operation) until complete isolation of the pulmonary veins was achieved (Figure 1F,G).

## 4 Postoperative Evaluation

After the procedure, the patient underwent another electrophysiological study. Atrial S1S1 programmed stimulation and intravenous isoproterenol infusion were used to test for arrhythmias. No arrhythmias were induced. There were no complications during the procedure. Postoperatively, the patient was prescribed amiodarone for 2 months and a novel oral anticoagulant for 3 months.

## 5 Outcome and Follow‐Up

The patient recovered smoothly without any complications. On the first postoperative day, a follow‐up electrocardiogram (ECG) showed normal heart rhythm with no signs of atrial fibrillation. A 24‐h Holter monitor was performed before discharge, revealing no abnormal rhythms. The patient has been followed up for over 6 months. During this period, ECG and Holter monitoring were conducted every 2 months or whenever palpitations occurred, along with regular telephone follow‐ups. No symptoms of tachycardia were reported, and there were no indications of tachycardia recurrence on either the ECG or Holter monitor during the follow‐up period.

## 6 Discussion

Dextrocardia is a rare congenital heart defect with an incidence of approximately 1 in 10,000. Some patients also present with anatomical abnormalities such as atrial septal defect, ventricular septal defect, valve anomalies, Tetralogy of Fallot, and transposition of the great arteries (Offen et al. 2016). In adult dextrocardia cases, the incidence of arrhythmias is about 50% (Zhang et al. 2022). Patients with dextrocardia are more prone to AF (Lim Jr. et al. 2023), and some may have abnormal pulmonary veins (Kohli and Hassan 2021). The anatomical characteristics of dextrocardia increase the difficulty of transseptal puncture and the complexity of radiofrequency ablation treatment.

The use of ICE allows for direct visualization of the transseptal puncture site, reducing the risk of complications and providing real‐time monitoring of the pericardium to ensure intraoperative safety (Asrress and Mitchell 2009; Dello Russo et al. 2010). In this case, we first performed a detailed assessment of the patient's cardiac structures using ICE, confirming the absence of other anatomical anomalies, and then proceeded with the transseptal puncture under ICE guidance.

The unique cardiac anatomy of patients with dextrocardia makes the identification of anatomical structures during ablation more challenging. The Carto 3 high‐density mapping system played a crucial role by providing three‐dimensional imaging to guide catheter manipulation, thus enhancing the accuracy and success rate of the ablation (Zhao et al. 2021). In patients with dextrocardia, catheter manipulation presents unique challenges due to the mirror‐image orientation of the heart structures. For example, in this case, we employed counterclockwise rotation of the intracardiac echocardiography (ICE) catheter from the right atrium to visualize the left atrium effectively. Additionally, although the posterior tilt on the ICE catheter remained useful, the reversed anatomy required adjustments in handling the catheter to maintain stable imaging and mapping. These modifications in catheter manipulation, facilitated by three‐dimensional imaging, were essential to successfully navigating the altered anatomy and ensuring precise targeting during the ablation procedure.

For this patient with mirror‐image dextrocardia, X‐ray guidance helped in catheter positioning. The combination of three‐dimensional imaging, X‐ray, and ICE further ensured the safety of the procedure.

## Author Contributions

**Shuang Zhang:** conceptualization, investigation, writing – original draft. **Mingxian Chen:** investigation, writing – review and editing. **Lin Hu:** investigation, writing – review and editing. **Hanze Tang:** investigation, writing – review and editing. **Liyi Liao:** investigation, writing – review and editing. **Xuping Li:** conceptualization, funding acquisition, resources, writing – review and editing.

## Consent

We confirm that informed consent was obtained from the patient in compliance with the Committee on Publication Ethics (COPE) guidelines. The patient was fully informed about the nature of the case report and agreed to the use of their clinical data and images for publication purposes. The patient's identity has been protected, and no identifiable information has been disclosed.

## Conflicts of Interest

The authors declare no conflicts of interest.

## Data Availability

The data that support the findings of this study are available from the corresponding author upon reasonable request.
